# Supplementary material for: Exosomes define a local and systemic communication network in healthy pancreas and pancreatic ductal adenocarcinoma
Source: Nat Commun. 2024 Feb 21;15:1496. doi: 10.1038/s41467-024-45753-7 (PMC10881969; doi:10.1038/s41467-024-45753-7)
Supplement: Supplementary file 1 — Supplementary Information [file 41467_2024_45753_MOESM1_ESM.pdf]

Supplementary Figure 1

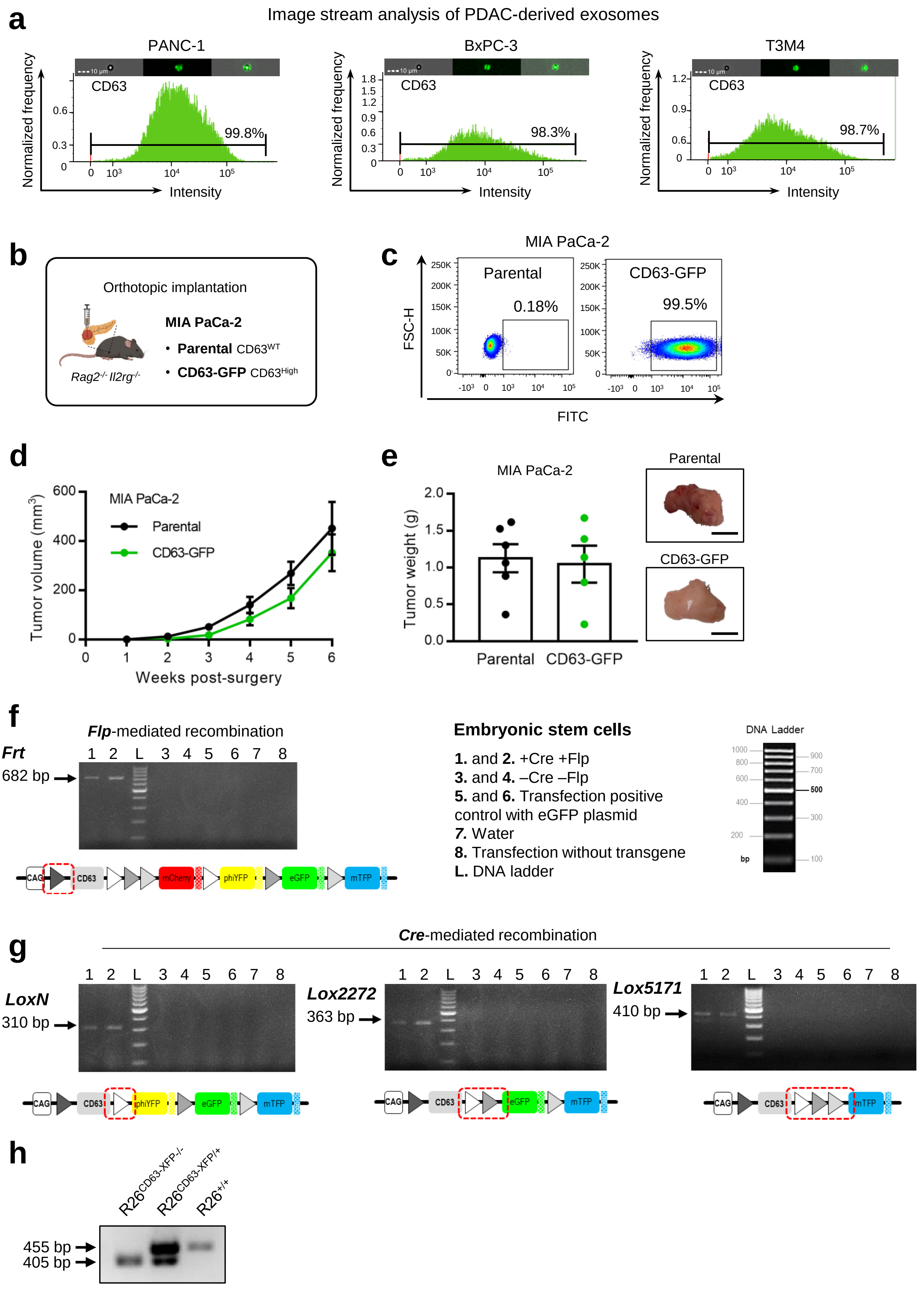

Supplementary Fig. 1 – CD63 is a good candidate for tagging pancreas-derived exosomes and the ExoBow transgene is efficiently recombined in pancreas cells. a Image stream analysis of CD63 in exosomes isolated from human pancreatic cancer cell lines: PANC-1, BxPC-3 and T3M4. Scale bar 10μm. **b** Experimental layout of MIA PaCa-2 CD63-GFP cell line and its parental counterpart orthotopically implanted in the pancreas of immunocompromised *Rag2*$^{-/-}$*Il2rg*$^{-/-}$ mice. **c** Representative FACS analysis of FITC signal in MIA PaCa-2 CD63-GFP cell line. MIA PaCa-2 parental was used as a negative control. **d** Growth curve measured by ultrasound of MIA PaCa-2 parental and MIA PaCa-2 CD63-GFP tumors. **e** Tumor weight (left) and representative tumor images at time of euthanasia (right) of MIA PaCa-2 parental (n=6) and MIA PaCa-2 CD63-GFP (n=5). Scale bar 1 cm. **f** Schematic representation of the assessed treatment conditions in embryonic stem cells by PCR. PCR gel depicting Flp-mediated recombination. **g** PCR gel depicting Cre-mediated recombination of LoxN sites (left panel), Lox2272 sites (middle panel) and Lox5171 sites (right panel). Arrows indicate PCR product in the expected size upon recombination. Control refers to embryonic stem cells transfected with eGFP plasmid (positive control for transfection). **h** Representative genotyping PCR of the ExoBow transgene (R26$^{CD63\text{-}XFP}$) in DNA derived from the ear of a R26$^{CD63\text{-}XFP-/-}$ homozygote mouse, R26$^{CD63\text{-}XFP/+}$ heterozygous mouse and WT mouse (R26$^{+/+}$). R26 WT allele 455 bp, R26$^{CD63\text{-}XFP}$ allele 405 bp. Data are Mean±SEM. Source data are provided as a Source Data file. Schemes created with BioRender.com.

Supplementary Figure 2

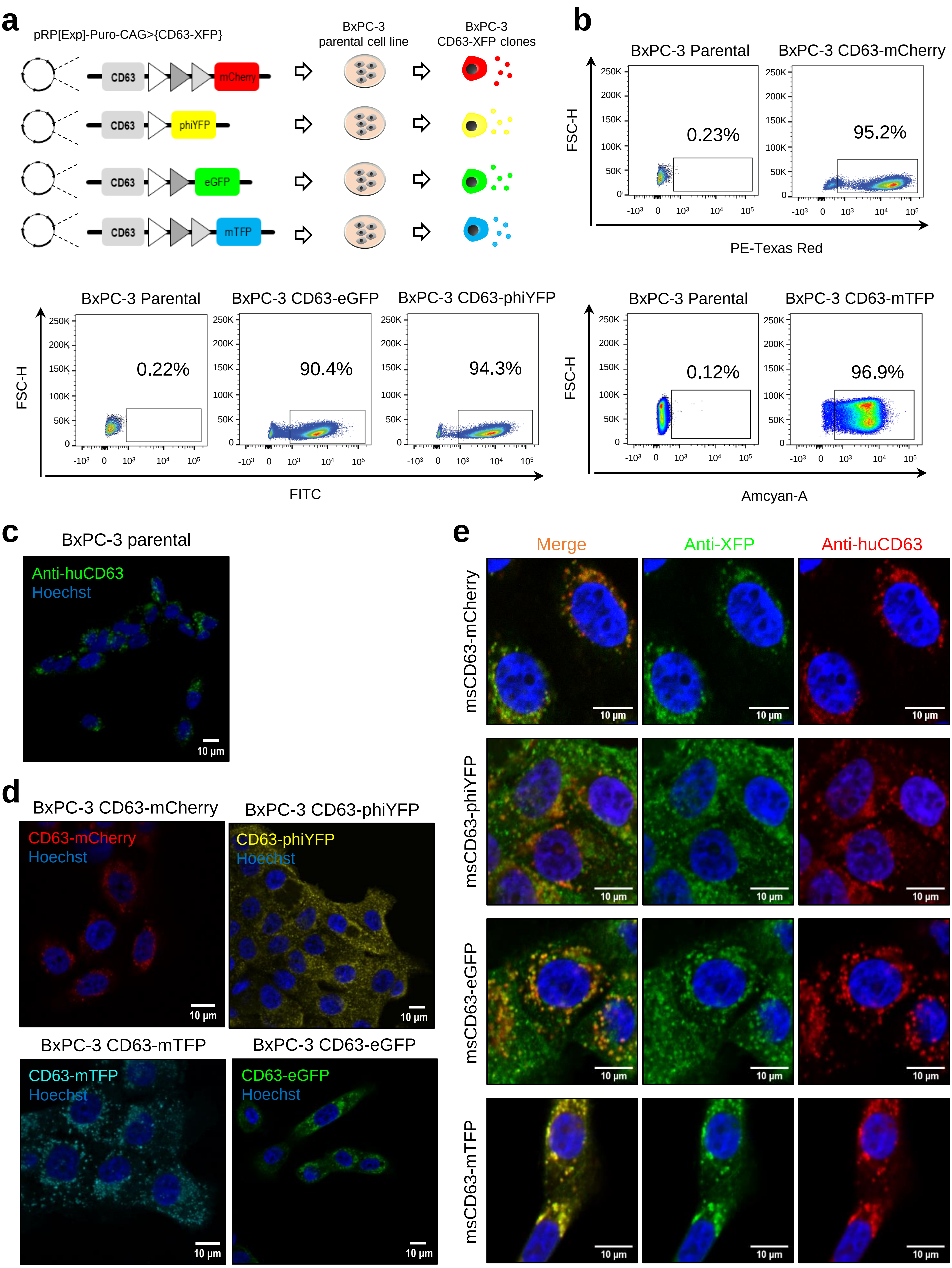

Supplementary Fig. 2 – CD63-XFP co-localizes with endogenous CD63 in PDAC cell lines. a Schematic representation of the cloning of each CD63-XFP (CD63 of mouse origin) sequence that results from the Flp and Cre mediated recombinations of the ExoBow transgene into the pRP[Exp]-Puro-CAG> plasmid and respective transfection into the human PDAC cell line, BxPC-3. **b** Representative FACS analysis of BxPC-3 CD63-XFP cell lines. PE-Texas Red channel was used to detect CD63-mCherry, FITC channel to detect CD63-eGFP and CD63-phiYFP and Amcyan-A channel to detect CD63-mTFP. BxPC-3 parental cell line was used as a negative control. **c** Confocal microscopy image of maximum projection of BxPC-3 parental cells immunostained against anti-human CD63 (green, endogenous CD63 of BxPC-3 parental cell line). **d** Confocal microscopy images of the fluorescent endogenous levels of each BxPC-3 CD63-XFP clone: CD63-mCherry (red), CD63-phiYFP (yellow), CD63-eGFP (green) and CD63-mTFP (cyan), using anti-XFP antibodies. **e** Confocal microscopy images of BxPC-3 CD63-XFP cells immunostained with respective anti-XFP antibody (green) and the anti-human CD63 antibody (red) to determine cellular localization. Nuclei counterstained with hoechst (blue). Scale bar 10μm. Source data are provided as a Source Data file.

Supplementary Figure 3

PDAC GEMMs CD63-XFP *ex vivo* cell lines

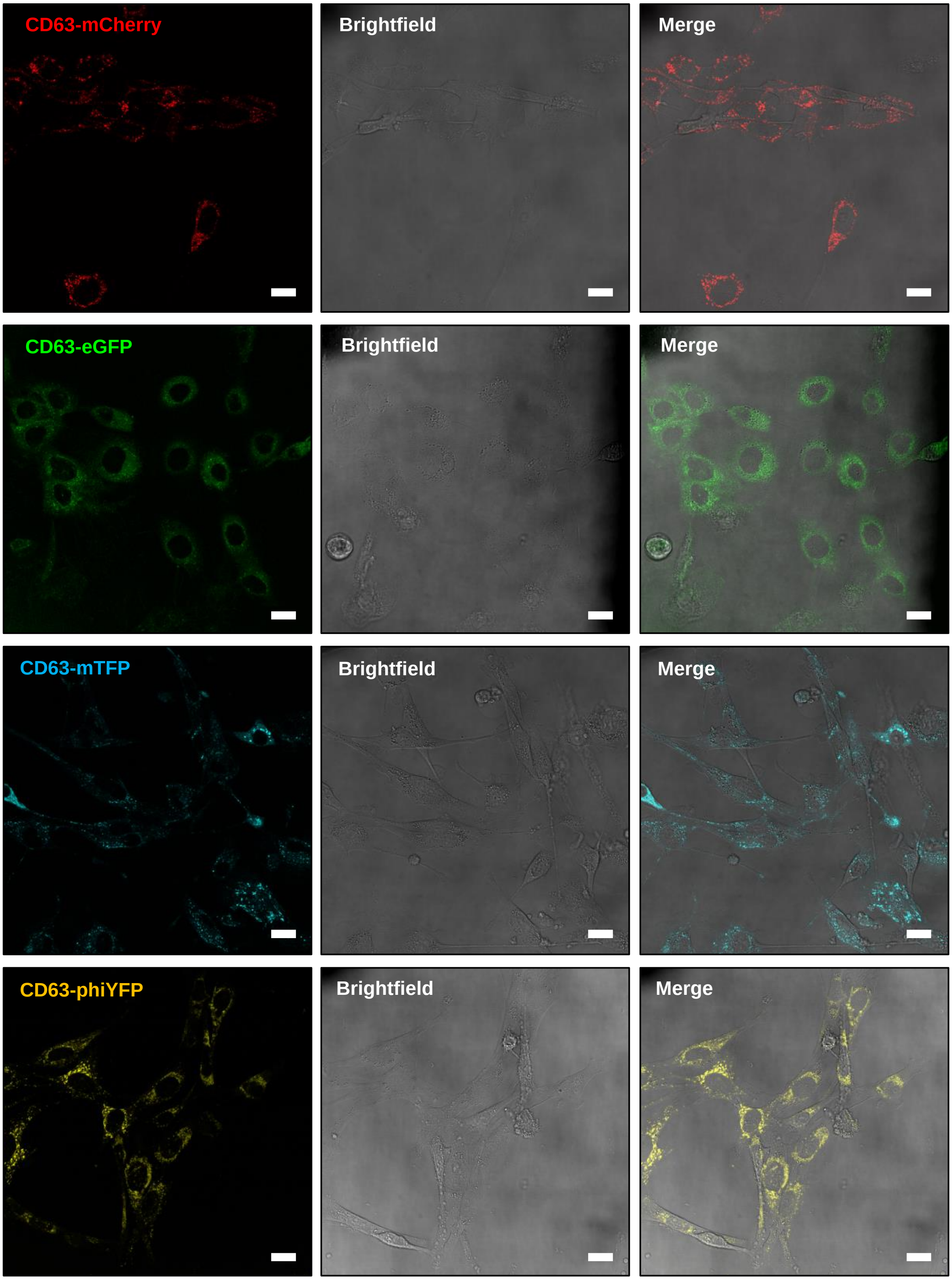

Supplementary Fig. 3 – CD63-XFP expression in cell lines established from reporter PDAC GEMMs – Confocal microscopic images of the fluorescent endogenous levels of CD63-mCherry (red), CD63-eGFP (green), CD63-mTFP (cyan) and CD63-phiYFP (yellow) in cell lines established from PDAC GEMMs expressing the ExoBow transgene. Scale bar 20µm.

Supplementary Figure 4

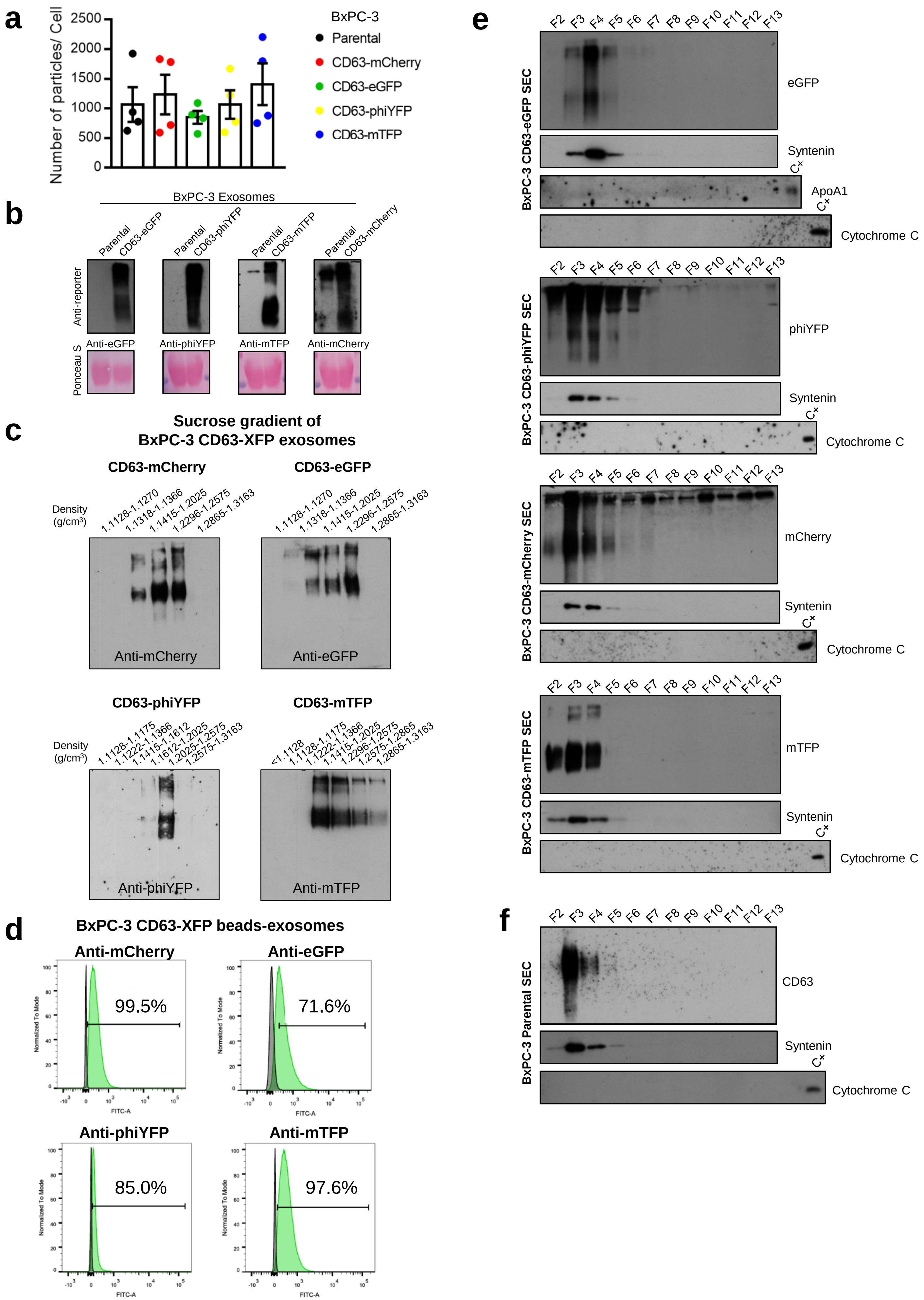

Supplementary Figure 4 – CD63-XFP PDAC cell lines produce color-coded exosomes. a Quantification of the number of particles normalized by the number of cells secreted by the each BxPC-3 clone measured by nanoparticle tracking assay. Data are Mean±SEM. **b** Western-blot against the reporter of exosomes isolated from each BxPC-3 CD63-XFP clone and its parental counterpart. Ponceau S was used as loading control. **c** Western-blot of EVs isolated from each BxPC-3 CD63-XFP clone by continuous sucrose gradient protocol. Individual 1 mL fractions were collected and after ultracentrifugation were loaded on gels for electrophoresis. Exosomes are present in fractions around 1.1415 to 1.2025 g/cm^3 density. Anti-mCherry, anti-eGFP, anti-phiYFP and anti-mTFP antibodies were used in the respective CD63-mCherry, CD63-eGFP, CD63-phiYFP and CD63-mTFP western-blots. **d** Representative histogram plots of FACS analysis of XFP-positive exosomes-coupled to beads derived from BxPC-3 CD63-XFP clones. Anti-mCherry, anti-eGFP, anti-phiYFP and anti-mTFP primary antibodies were used for exosomes isolated from the respective BxPC-3 CD63-mCherry, CD63-eGFP, CD63-phiYFP and CD63-mTFP cell lines followed by incubation with a secondary antibody tagged with AF488. Exosomes-coupled to beads stained only with secondary antibody were used as negative control. **e** Western-blot of EVs isolated from each BxPC-3 CD63-XFP clone by size-exclusion chromatography (SEC). Anti-mCherry, anti-eGFP, anti-phiYFP and anti-mTFP antibodies were used in the respective CD63-mCherry, CD63-eGFP, CD63-phiYFP and CD63-mTFP western-blots. Syntenin was used as an exosomal marker. ApoA1 and cytochrome C were used to demonstrate the purity of the EVs preparations. **f** CD63 western-blot of EVs isolated from the BxPC-3 parental cell line by SEC. Syntenin was used as an exosomal marker. Cytochrome C was used to demonstrate the purity of the EVs preparations. Source data are provided as a Source Data file.

Supplementary Figure 5

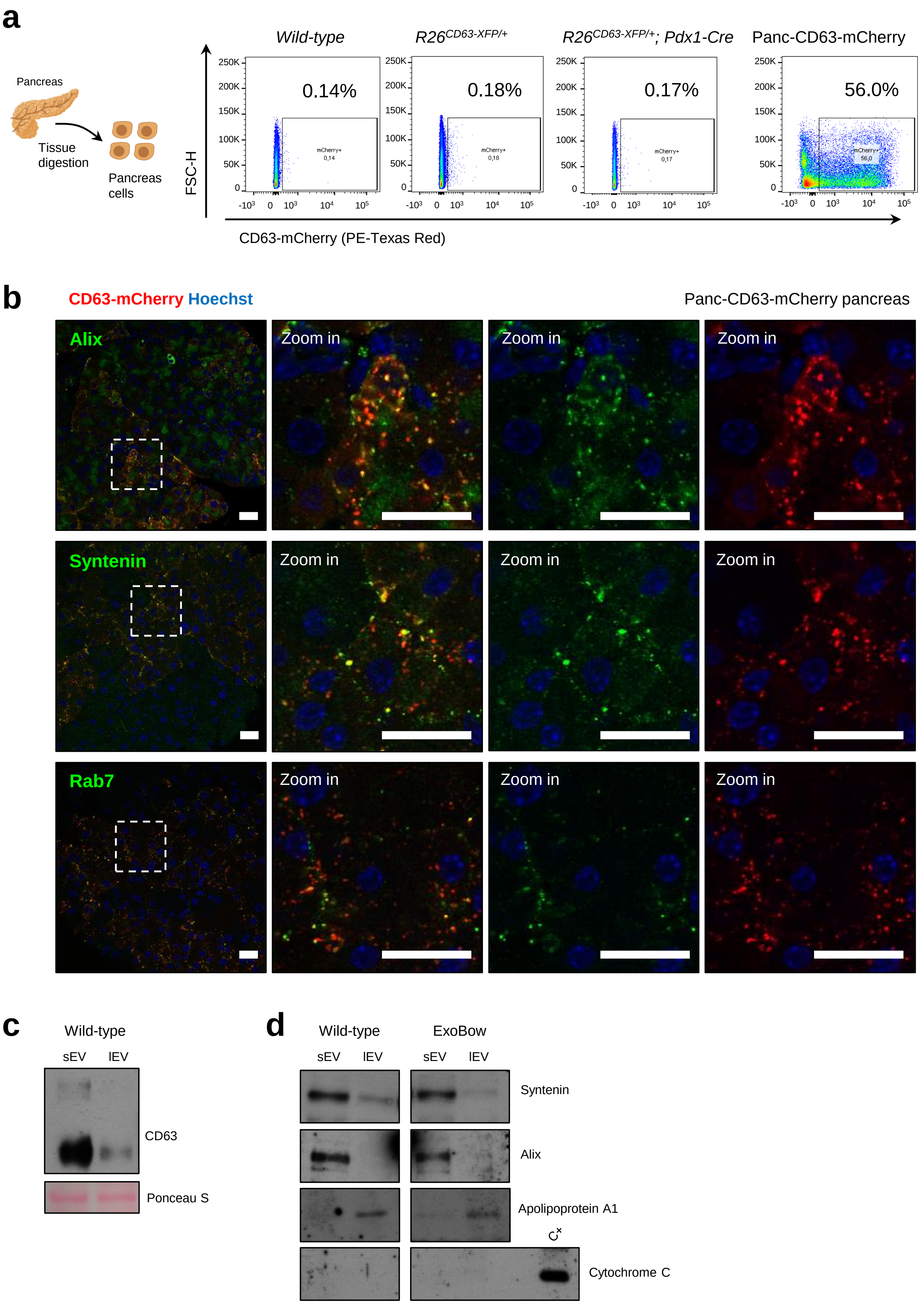

Supplementary Figure 5 – Panc-CD63-mCherry model efficiently expresses CD63-mCherry in pancreas cells which co-localizes with endosomal markers. a Schematic depicting pancreas digestion for single cell analysis by flow cytometry (left) and representative flow cytometry plots (right) of CD63-mCherry positive cells in a pancreas of a Panc-CD63-mCherry mouse in comparison to the pancreas of a wild-type (WT), *R26*$^{CD63\text{-}XFP/+}$, and *R26*$^{CD63\text{-}XFP/+}$*; Pdx1-Cre* control mice. **b** Confocal microscopic images of maximum projection of a pancreas from Panc-CD63-mCherry mouse immunostained for Alix, Syntenin or Rab7 (green). Endogenous levels of CD63-mCherry and nuclei counterstained with hoechst are depicted in red and blue, respectively. Dashed lines identify zoom-in areas. Scale bar 20μm. **c** CD63 western-blot in small EVs (sEV) and large EVs (lEV) isolated from the pancreas of a WT mouse (control). Ponceau S was used as loading control. **d** Western-blot characterization of small EVs (sEV) and large EVs (lEV) isolated from the pancreas of WT (control) and ExoBow mice. Syntenin and Alix were used as exosomal markers. ApoA1 and cytochrome C were used to demonstrate the purity of the EVs preparations. Source data are provided as a Source Data file. Schemes created with BioRender.com.

Supplementary Figure 6

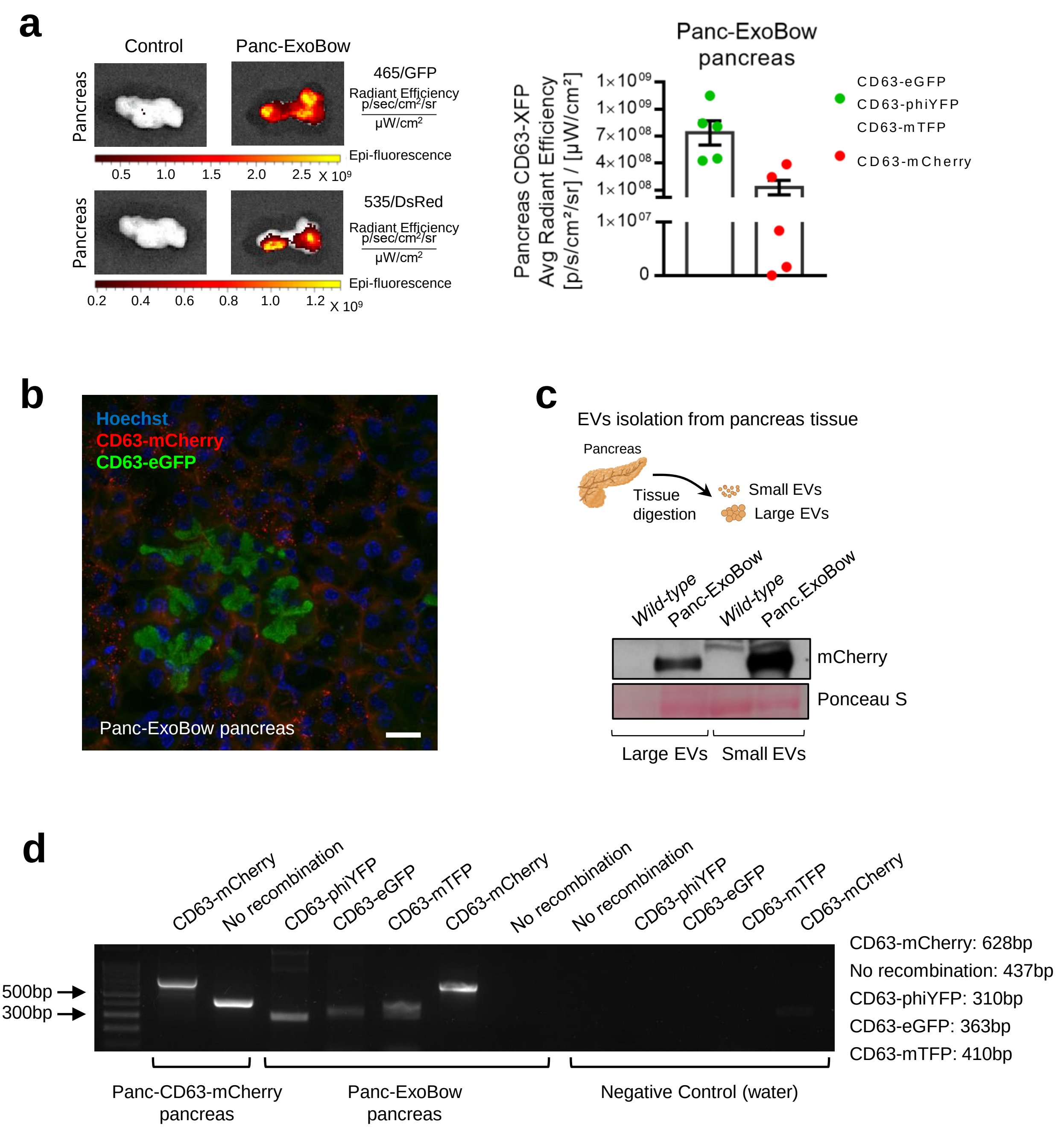

Supplementary Fig. 6 – Reporter mice pancreas cells efficiently express CD63-XFP and produce color-coded exosomes. a Pancreas imaging using IVIS Lumina System illustrating CD63-mCherry (535 excitation laser and DsRed emission filter) and CD63-eGFP, CD63-phiYFP, and CD63-mTFP (465 excitation laser and GFP emission filter). Pancreas of a control ($R26^{CD63\text{-}XFP/+,}$ no recombinases, left) and a multireporter mouse model Panc-ExoBow (right). Graphical representation depicting the average radiant efficiency fluorescence levels of CD63-XFP in pancreas of Panc-ExoBow mice (n=5). Data are Mean±SEM. **b** Confocal images of a pancreas section depicting CD63-eGFP (green) and CD63-mCherry (red) positive cells in the Panc-ExoBow model. Immunofluorescence for eGFP and mCherry and nuclei were counterstained with hoechst (blue). Scale bar 20µm. **c** Schematic representation of the isolation of interstitial EVs from the pancreas tissue by ultracentrifugation according to Crescitelli *et al*.[56]. Anti-mCherry western-blot in small and large EVs fractions isolated from pancreas tissue of *wild-type* (WT, control) or Panc-ExoBow mice. 25µg of protein samples used, Ponceau S was used as loading control. **d** PCR gel depicting Flp-mediated and Cre-mediated recombination of LoxN sites, Lox2272 sites and Lox5171 sites in pancreas samples of Panc-CD63-mCherry and Panc-ExoBow mice. Source data are provided as a Source Data file. Schemes created with BioRender.com.

Supplementary Figure 7

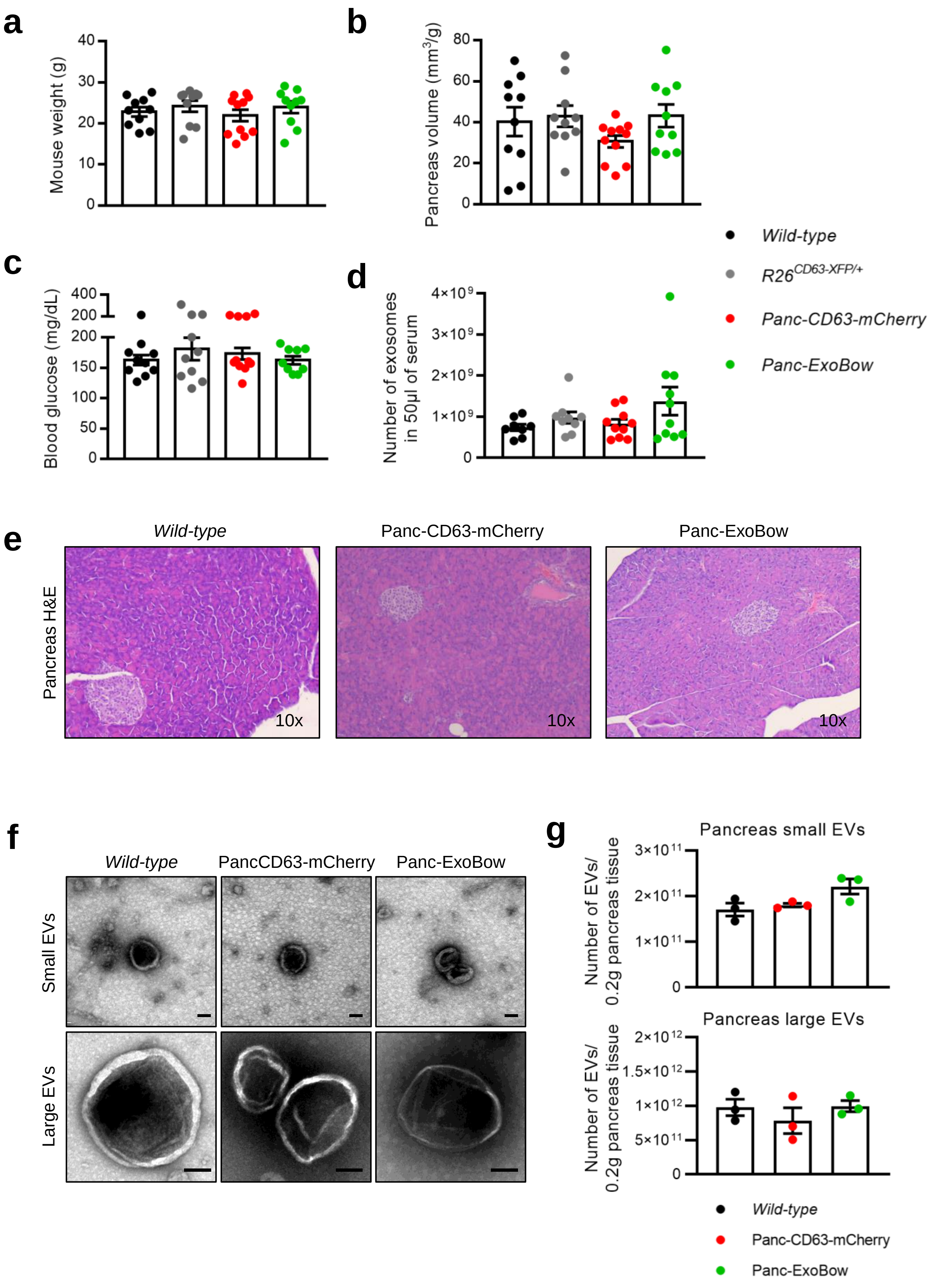

Supplementary Fig. 7 – CD63 expression does not impact mice physiology. Mouse weight **a** pancreas volume normalized by mouse weight **b**, blood glucose levels **c** and number of exosomes found in serum quantified by nanoparticle tracking assay **d** of *wild-type* (WT, n=10, except for serum exosomes n=8), $R26^{CD63\text{-}XFP/+}$ (n=10, except for serum exosomes n=9), Panc-CD63-mCherry (n=11, except for serum exosomes n=10) and Panc-ExoBow mice (n=10, except for blood glucose n=9). For serum exosomes concentration outlier detection was based on ROUT method. **e** Representative photos of H&E staining of WT, Panc-CD63-mCherry and Panc-ExoBow mice pancreas (10x). **f** Representative photos of WT, Panc-CD63-mCherry and Panc-ExoBow mice pancreas small EVs (upper panel, scale bar 50nm) and large EVs (lower panel, scale bar 100nm) by transmission electron microscopy. **g** Nanoparticle tracking analysis of small EVs (upper panel) and large EVs (lower panel) per 0.2g of pancreas tissue isolated from WT (n=3), Panc-CD63mCherry (n=3) and Panc-ExoBow (n=3) mice. Data are Mean±SEM.

Supplementary Figure 8

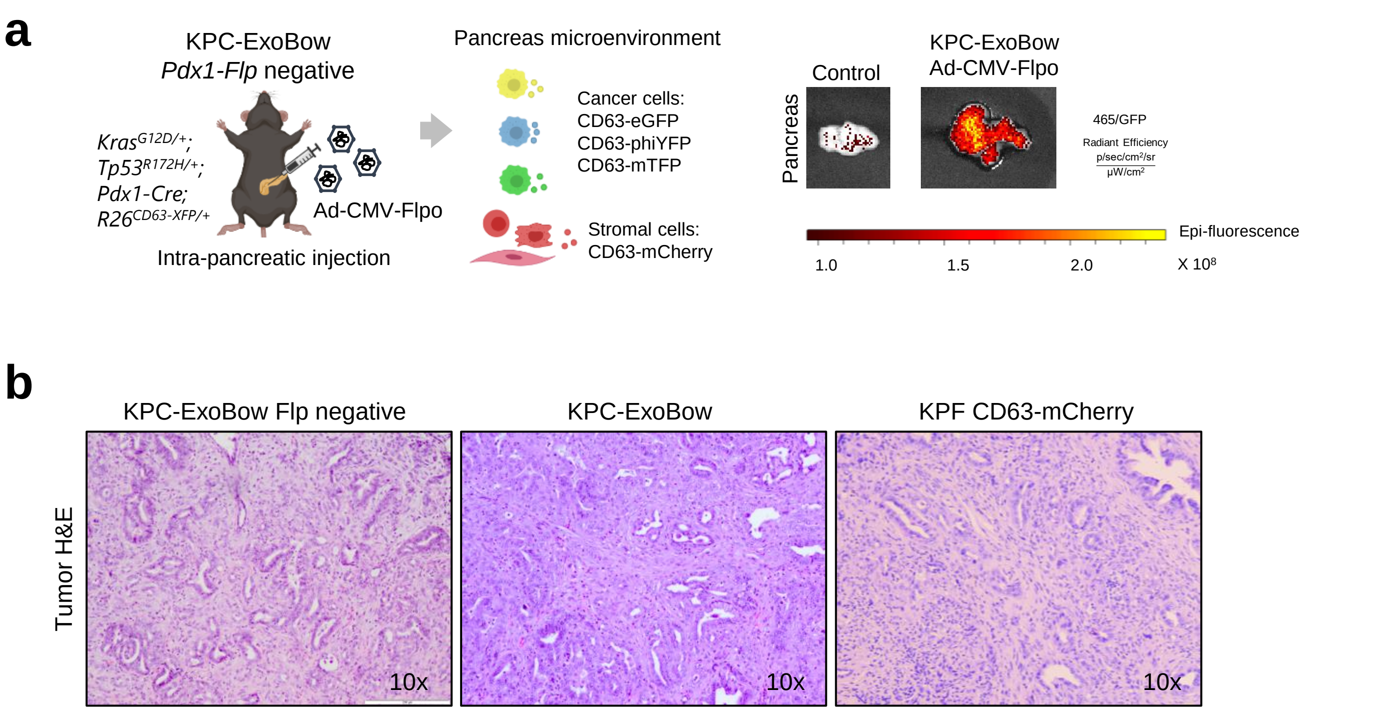

Supplementary Fig. 8 – CD63-XFP expression does not impact PDAC histology. a Experimental approach of intra-pancreatic injection of Ad-CMV-Flpo in KPC-ExoBow Flp negative mice (left) with representative IVIS image of a control *wild-type* pancreas and KPC-ExoBow Ad-CMV-Flpo tumor at euthanasia (right, 465 laser and GFP emission filter). **b** Representative photos of H&E staining of KPC-ExoBow Flp negative mice, KPC-ExoBow and KPF-CD63-mCherry mice pancreas (10x). Schemes created with BioRender.com.

Supplementary Figure 9

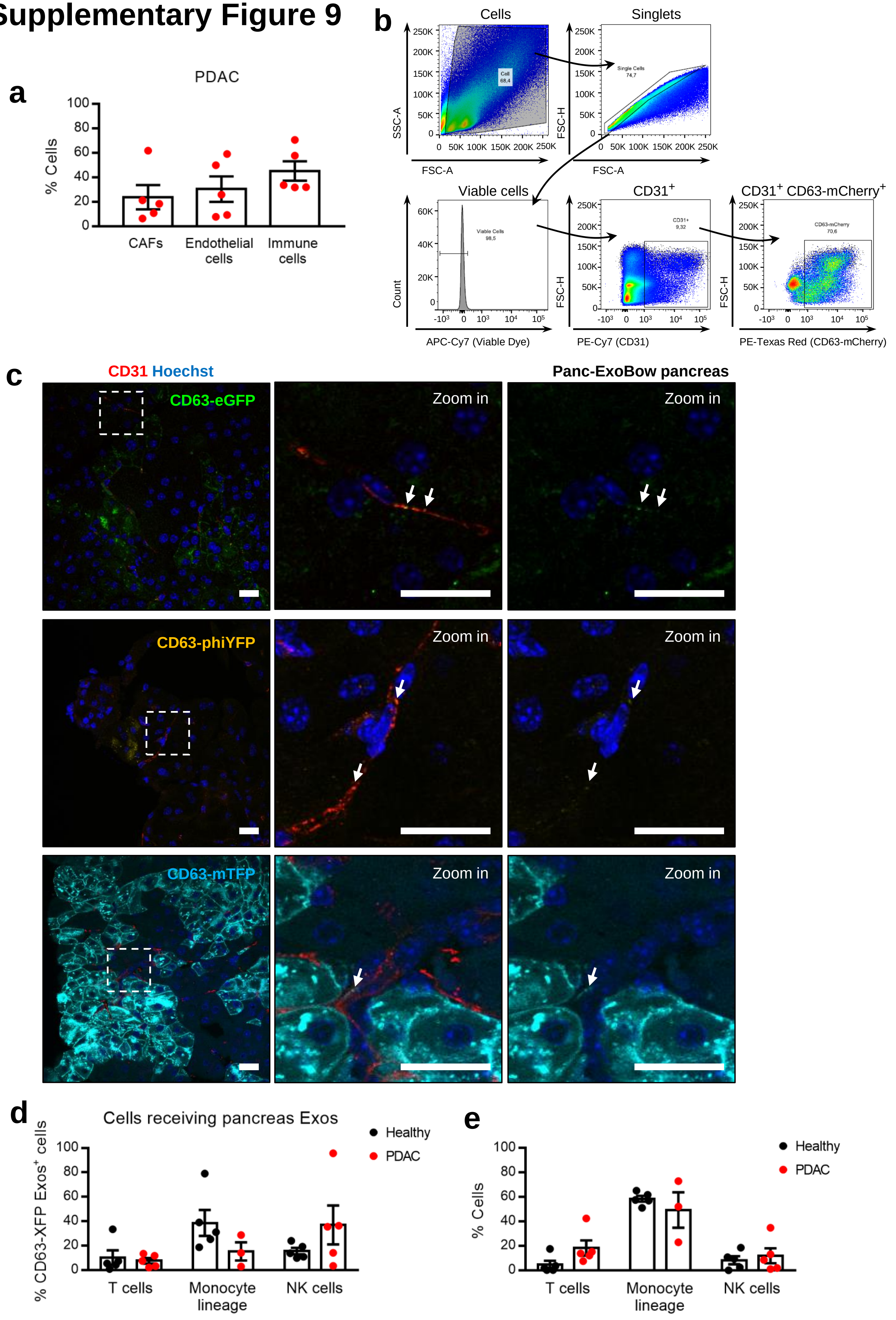

Supplementary Fig. 9 – Exosomes mediate intra-pancreas communication in PDAC. a Dot plot representing the percentage of cancer-associated fibroblasts (CAFs, CD140A$^+$), endothelial cells (CD31$^+$) and immune cells (CD45$^+$) in tumors of KPF CD63-mCherry (n=2) and KPC-ExoBow mice (n=3) analyzed by flow cytometry. **b** Related to figure 3a: representative plots for KPF CD63-mCherry tumor illustrating the gating strategy for flow cytometry analysis of endothelial cells receiving CD63$^+$PDAC Exos. FSC/SSC gate to identify cells, FSC-H/FSC-A to identify single cells types; APC-Cy7 negative gate for viable cells, PE-Cy7 for CD31 positive cells and PE-Texas Red for CD63-mCherry positive cells. Gates were defined using controls: unstained reporter tumor samples and stained non-ExoBow reporter tumor samples. A similar gating strategy was applied for the analysis of CAFs (CD140A$^+$) and immune cells (CD45$^+$). **c** Representative confocal microscopy images of pancreas CD63-XFP$^+$ Exos (CD63-eGFP - green, CD63-phiYFP - yellow and CD63-mTFP - cyan) accumulation in endothelial cells (CD31, red). Nuclei were counterstained with hoechst (blue). Scale bar 20µm. **d** Dot plot representing the percentage of T cells (CD3$^+$), cells of the monocyte lineage (CD45$^+$CD11b$^+$Ly6G/C$^-$) and natural killer (NK, CD3$^-$NK$^+$) cells that received pancreas CD63$^+$ Exos in healthy (n=5) and in PDAC (n=5, except for monocyte-lineage: n=3 KPC-ExoBow). Gating strategy included in Source Data file. **e** Dot plot representing the percentage of T cells, cells of the monocyte lineage and NK cells in the healthy pancreas microenvironment (Panc-CD63-mCherry n=5) and in PDAC (n=5, except for monocyte-lineage: n=3 KPC-ExoBow). Gating strategy included in Source Data file. Data are Mean±SEM.

Supplementary Figure 10

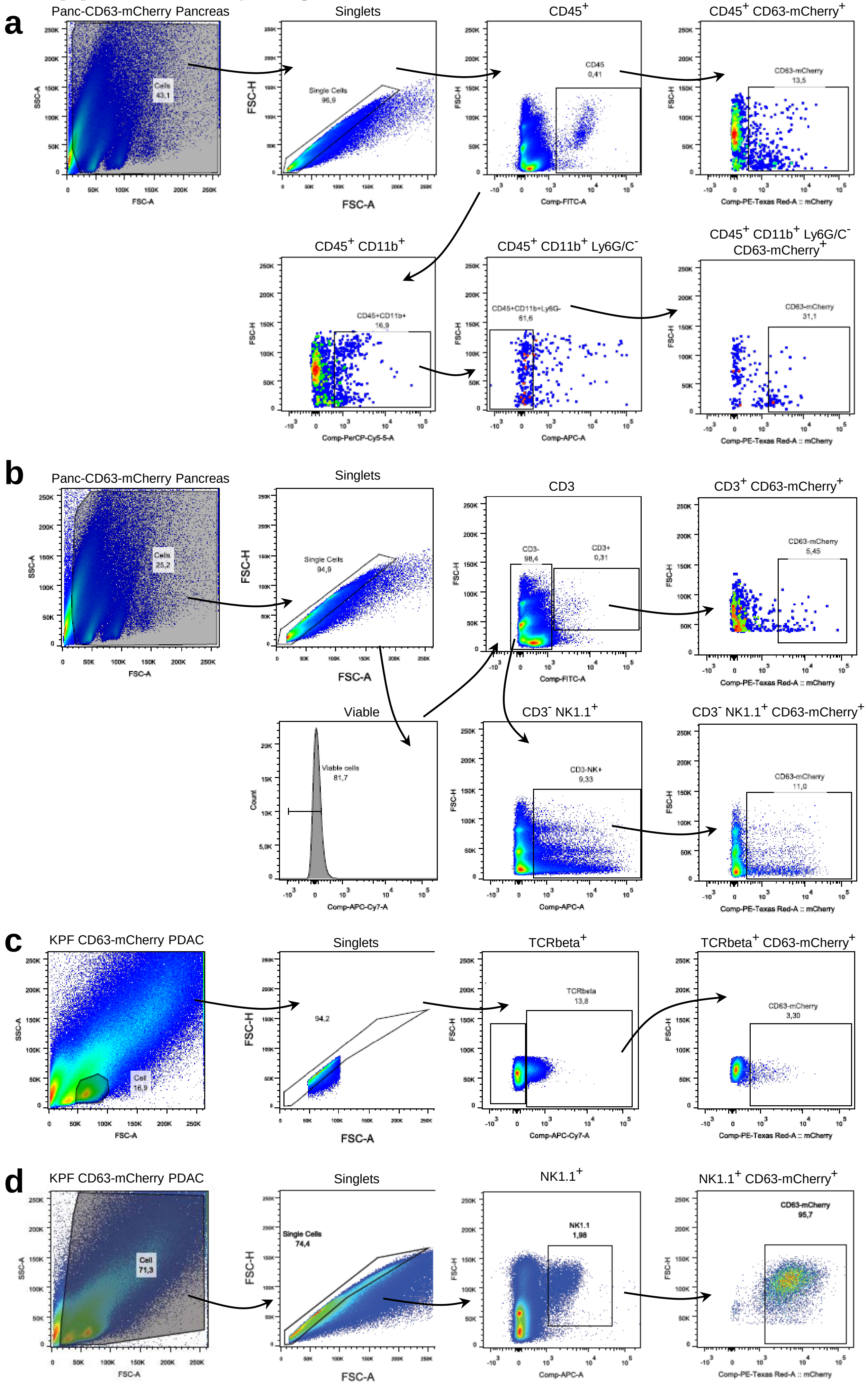

Supplementary Fig. 10 – Exosomes mediate intra-pancreas communication in health and in PDAC. (Related to Supplementary Figure 9). **a** Representative plots for Panc-CD63-mCherry pancreas illustrating the gating strategy for flow cytometry analysis of immune cells of the monocyte lineage ($CD45^+CD11b^+Ly6G/C^-$) receiving $CD63^+$ Exos: FSC/SSC gate to identify cells, FSC-H/FSC-A to identify single cells types, FITC for CD45 positive cells, PerCP-Cy5.5 for CD11b, APC for Ly6G/C, and PE-Texas Red for CD63-mCherry positive cells. **b** Representative plots for Panc-CD63-mCherry pancreas illustrating the gating strategy for flow cytometry analysis of T cells ($CD3^+$) and Natural Killer (NK, $CD3^-NK1.1^+$) cells receiving $CD63^+$ Exos: FSC/SSC gate to identify cells, FSC-H/FSC-A to identify single cells types, APC-Cy7 negative gate for viable cells, FITC for CD3 positive cells, APC for NK1.1 (inside CD3 negative gate) and PE-Texas Red for CD63-mCherry positive cells. **c** Representative plots for KPF CD63-mCherry tumor illustrating the gating strategy for flow cytometry analysis of T cells ($TCR\beta^+$) receiving $CD63^+$ Exos: FSC/SSC gate to identify cells, FSC-H/FSC-A to identify single cells types, APC-Cy7 for TCRβ positive cells and PE-Texas Red for CD63-mCherry positive cells. **d** Representative plots for KPF CD63-mCherry tumor illustrating the gating strategy for flow cytometry analysis of NK cells ($NK1.1^+$) receiving $CD63^+$ Exos: FSC/SSC gate to identify cells, FSC-H/FSC-A to identify single cells types, APC for NK positive cells and PE-Texas Red for CD63-mCherry positive cells. In all cases gates were defined using controls: unstained reporter pancreas/tumor samples and stained non-ExoBow reporter pancreas/tumor samples. Additional gating strategy has been included in Source Data file.

Supplementary Figure 11

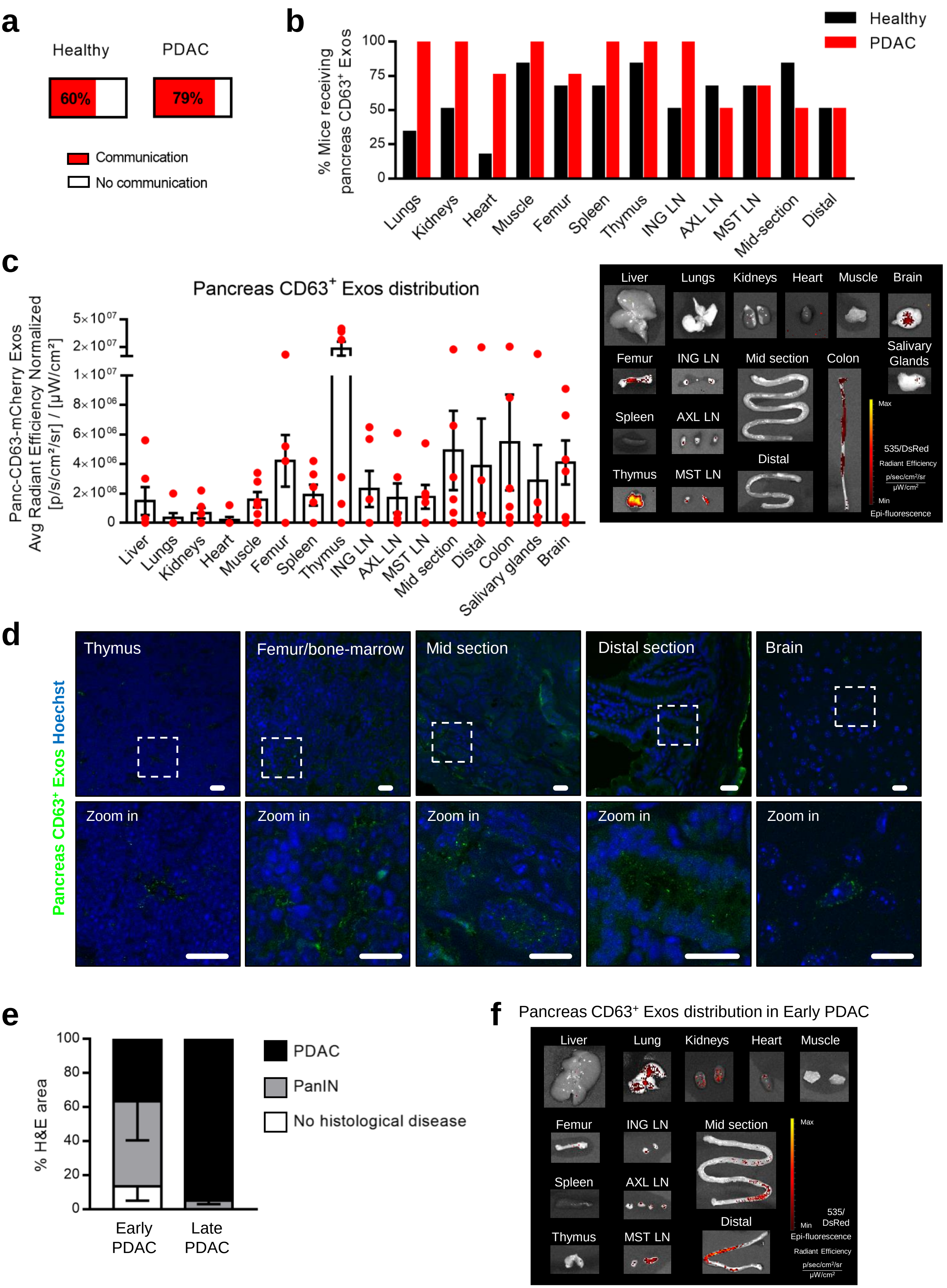

Supplementary Fig. 11 – Exosomes mediate pancreas inter-organ connectome in health and cancer. **a** Percentage of organs that received (Communication) or did not receive (No communication) pancreas CD63-mCherry$^+$ Exos in health (Panc-CD63-mCherry, n=6) and in PDAC (KPF CD63-mCherry, n=4). **b** Bar graph representing the percentage of mice that were positive for pancreas CD63-mCherry$^+$ Exos in each organ in healthy (Panc-CD63-mCherry, n=6) and in PDAC (KPF CD63-mCherry, n=4). **c** Dot plot representing the average radiant efficiency fluorescence levels of pancreas CD63-mCherry$^+$ Exos across all organs in Panc-CD63-mCherry mice (n=6) at time of euthanasia (left), with representative IVIS images (535 excitation laser and DsRed emission filter; right). **d** Representative confocal microscopy images of pancreas CD63$^+$ Exos (green) accumulation in the thymus, femur/bone-marrow, intestinal mid-section and distal parts and brain of Panc-CD63-mCherry mice. Nuclei were counterstained with hoechst (blue). Scale bar 20μm. **e** Percentage of no histological disease, PanIN and PDAC area in tumors of mice grouped in early (KPF CD63-mCherry n=1 and KPC-ExoBow Adeno-Flpo n=2) and late (KPF CD63-mCherry n=4) PDAC stages. **f** Representative IVIS images (535 excitation laser and DsRed emission filter) of radiant efficiency fluorescence of pancreas CD63-mCherry$^+$ Exos across all organs in KPF CD63-mCherry at an early PDAC stage. Data are Mean±SEM. ING LN, inguinal lymph nodes; AXL LN, axillary lymph nodes; MST LN, mesenteric lymph nodes; PanIN, Pancreatic Intraepithelial Neoplasia.

Supplementary Figure 12

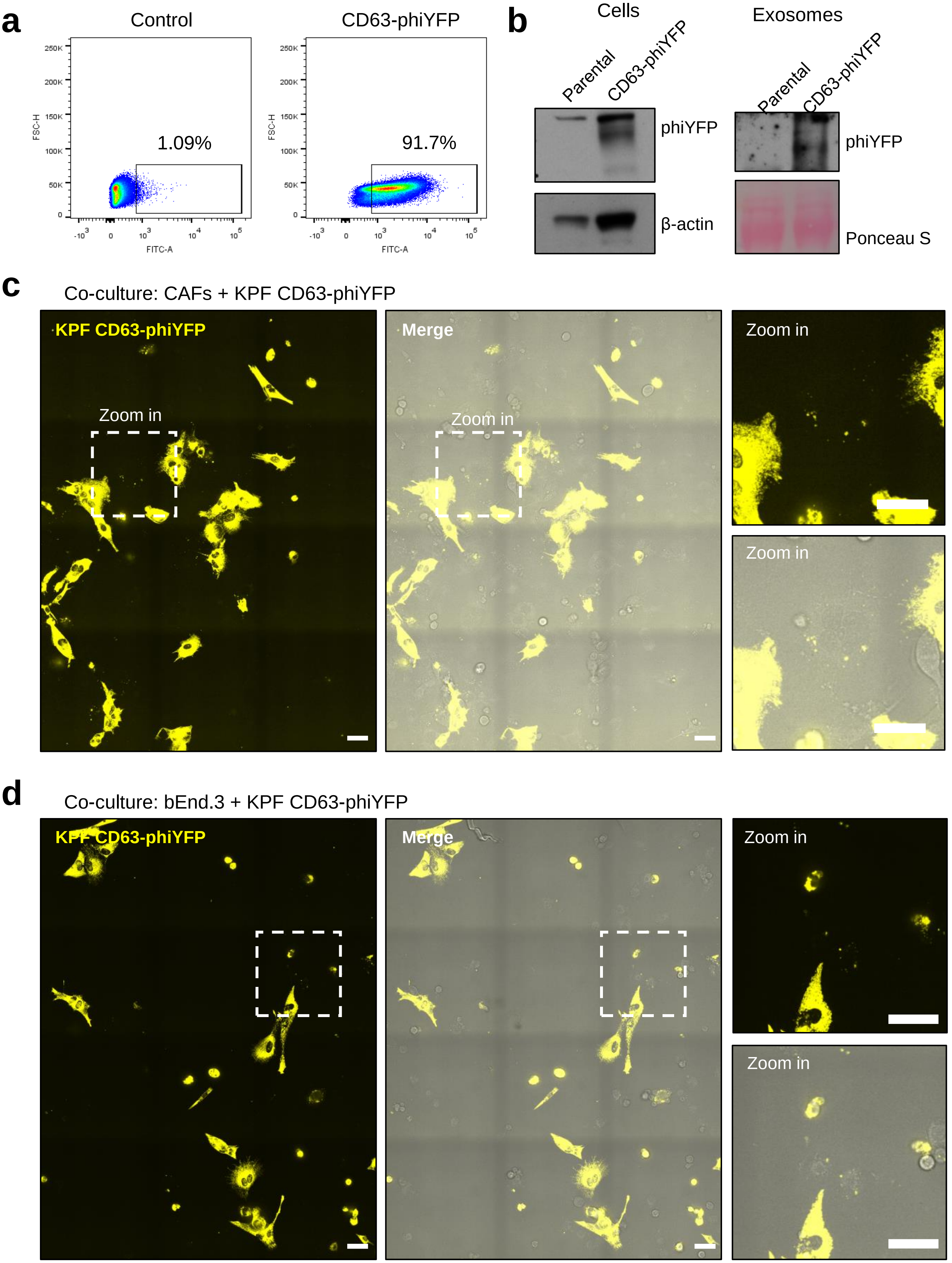

Supplementary Fig. 12 – CD63-phiYFP PDAC exosomes are taken up by cancer associated fibroblasts and endothelial cells. a Representative FACS analysis of FITC (CD63-phiYFP) signal in KPF CD63-phiYFP cell line. A KPC cell line was used as a negative control. **b** Anti-phiYFP western-blot of cells and respective ultracentrifuged isolated exosomes. β-Actin and Ponceau S were used as loading controls. Representative microscopy tile scan images of co-cultures of KPF CD63-phiYFP with **c** CAFs or **d** bEnd.3 endothelial cells. Scale bar 50μm.
