# Supplementary material for: Exosomes define a local and systemic communication network in healthy pancreas and pancreatic ductal adenocarcinoma
Source: Nat Commun. 2024 Feb 21;15:1496. doi: 10.1038/s41467-024-45753-7 (PMC10881969; doi:10.1038/s41467-024-45753-7)
Supplement: Supplementary file 2 — Reporting Summary [file 41467_2024_45753_MOESM2_ESM.pdf]

nature portfolio

Corresponding author(s): Sonia A. Melo

Last updated by author(s): 13/12/23

Reporting Summary

Nature Portfolio wishes to improve the reproducibility of the work that we publish. This form provides structure for consistency and transparency in reporting. For further information on Nature Portfolio policies, see our Editorial Policies and the Editorial Policy Checklist.

Statistics

For all statistical analyses, confirm that the following items are present in the figure legend, table legend, main text, or Methods section.

n/a	Confirmed	
☐	☒	The exact sample size (*n*) for each experimental group/condition, given as a discrete number and unit of measurement
☐	☒	A statement on whether measurements were taken from distinct samples or whether the same sample was measured repeatedly
☐	☒	The statistical test(s) used AND whether they are one- or two-sided<br>*Only common tests should be described solely by name; describe more complex techniques in the Methods section.*
☒	☐	A description of all covariates tested
☒	☐	A description of any assumptions or corrections, such as tests of normality and adjustment for multiple comparisons
☐	☒	A full description of the statistical parameters including central tendency (e.g. means) or other basic estimates (e.g. regression coefficient) AND variation (e.g. standard deviation) or associated estimates of uncertainty (e.g. confidence intervals)
☐	☒	For null hypothesis testing, the test statistic (e.g. *F*, *t*, *r*) with confidence intervals, effect sizes, degrees of freedom and *P* value noted<br>*Give P values as exact values whenever suitable.*
☒	☐	For Bayesian analysis, information on the choice of priors and Markov chain Monte Carlo settings
☒	☐	For hierarchical and complex designs, identification of the appropriate level for tests and full reporting of outcomes
☒	☐	Estimates of effect sizes (e.g. Cohen's *d*, Pearson's *r*), indicating how they were calculated

Our web collection on statistics for biologists contains articles on many of the points above.

Software and code

Policy information about availability of computer code

Data collection	Ex-vivo fluorescence imaging was obtained using IVIS Lumina iii (Caliper).<br>Tumor growth was monitored by ultrasound (Micro Ultrasound Vevo 2100).<br>Flow cytometry data were collected using BD FACS Aria II Cell Sorter, LSR Fortessa and BD FACSCANTO II (BD Biosciences).<br>Immunofluorescence images were acquired using a Leica TCS SP5 inverted confocal and an upright Zeiss LSM 780.<br>Co-culture immages were acquired using a CFI Plan Apo VC 60XC WI /1.2 objective on a Nikon ECLIPSE Ti2 microscope equipped with a CrestOptics X-Light V3 spinning disk and a Photometrics sCMOS Kinetix camera.<br>TEM images were taken by JEOL JEM 1400 TEM at 120 kV.<br>Extracellular vesicles were analyzed using nanoparticle tracking analysis (NTA) (NanoSight NS300) and ImageStreamx MarkII Imaging Flow Cytometry (Luminex Amnis Image Stream Multispectral Imaging Flow Cytometer).<br>RNA Sequencing was performed on an Ion S5XL™ sequencer (Thermo Fisher Scientific).<br>Protein identification and quantitation was performed by nanoLC-MS/MS equipped with a Field Asymmetric Ion Mobility Spectrometry - FAIMS interface. The system is composed of a Vanquish Neo liquid chromatography system coupled to an Eclipse Tribrid Quadrupole, Orbitrap, Ion Trap mass spectrometer (Thermo Fisher Scientific, San Jose, CA).<br>Proteomics raw data was processed using the Proteome Discoverer 3.0.1.27 software (Thermo Fisher Scientific).
Data analysis	Statistical analysis was performed using GraphPad Prism® (v7.0).<br>The biodistribution of fluorescent extracellular vesicles was analyzed with the IVIS Living image software (v4.3.1) .<br>Flow cytometry data was analyzed using FlowJo software (v10, BD).<br>ImageStream data was analyzed using INSPIRE (V4.1.426.0).<br>Immunofluorescence images were analyzed with the software Fiji/ImageJ Java (v1.8.0).

Proteomics and RNA Seq analysis were done using R (v4.1.2):
Proteomics : R packages MsnBase (v2.20.4);
RNAseq: R packages Deseq2 (v1.34.0) and DESeq (v1,12,1; CAFs and bEnd.3);
Pathway analysis: ClusterProfiler (v4.2.2);
Plots: ComplexHeatmap (v2.10.0), ggplot2(v3.4.0), circlize (v 0.4.15).

For manuscripts utilizing custom algorithms or software that are central to the research but not yet described in published literature, software must be made available to editors and reviewers. We strongly encourage code deposition in a community repository (e.g. GitHub). See the Nature Portfolio guidelines for submitting code & software for further information.

Data

Policy information about availability of data

All manuscripts must include a data availability statement. This statement should provide the following information, where applicable:

- Accession codes, unique identifiers, or web links for publicly available datasets
- A description of any restrictions on data availability
- For clinical datasets or third party data, please ensure that the statement adheres to our policy

The data supporting the findings of this study are available within the paper and its' Supplementary Information. Figures data have associated raw data included in Source Data file: Figures 1-7 and Supplementary Figures 1-9,11,12. The mass spectrometry proteomics data have been deposited to the ProteomeXchange Consortium via the PRIDE partner repository with the dataset identifier PXD047009 (https://www.ebi.ac.uk/pride/archive/projects/PXD047009). RNA Seq data generated within this study has been submitted to European Nucleotide Archive (ENA) browser under the accession code PRJEB71061 (https://www.ebi.ac.uk/ena/browser/view/PRJEB71061). All other relevant data supporting the key findings of this study are available as Supplementary Information files (Supplementary Figures 1-12, and Source Data file) or from the corresponding author upon reasonable request. No data availability restrictions.

Research involving human participants, their data, or biological material

Policy information about studies with human participants or human data. See also policy information about sex, gender (identity/presentation), and sexual orientation and race, ethnicity and racism.

Reporting on sex and gender	N/A
Reporting on race, ethnicity, or other socially relevant groupings	N/A
Population characteristics	N/A
Recruitment	N/A
Ethics oversight	N/A

Note that full information on the approval of the study protocol must also be provided in the manuscript.

Field-specific reporting

Please select the one below that is the best fit for your research. If you are not sure, read the appropriate sections before making your selection.

☒ Life sciences ☐ Behavioural & social sciences ☐ Ecological, evolutionary & environmental sciences

For a reference copy of the document with all sections, see nature.com/documents/nr-reporting-summary-flat.pdf

Life sciences study design

All studies must disclose on these points even when the disclosure is negative.

Sample size	For in vivo studies, n=3-8 mice per group was sufficient to detect meaningful biological differences with good reproducibility. No statistical methodology was calculated in this study to determine the necessary sample size. However, our choice on the replicate number was based on our previous studies (Ruivo, C.F. and Bastos, N. et. al., PMID: 35012996) as well as previous publications which had statistical significance observations to similar experimental approaches (Men, Y. et. al., PMID: 31515491; Luo, W. et. al. 2020, PMID: 32157172). Furthermore, the replicate numbers were based on the relative variance in the assay.
Data exclusions	Some organs were excluded from the biodistribution analysis due to known promoter leakage since it represented a bias for the interpretation of the biological role of extracellular vesicles in vivo, as explained in detail in the manuscript. Some mice were found as outliers in the number of blood exosomes analysis as stated in the figure legend.
Replication	In this study we have generated a new mouse from which the experimental outcome can be reliably observed. In addition, we have repeated all experimental findings sufficiently, as described in the methods and figure legends.
Randomization	No randomization required in the present study since no treatment conditions were compared. All comparisons were performed between

Randomization	different genotypes which do not require randomization.
Blinding	The investigators were blinded to the design of the study in the following described cases. During injections of tumor cells in the mouse pancreas, the surgeon was blinded to the experimental conditions. In immunohistochemistry scoring, operators were blinded to the groups of the animals being analyzed. Other than the abovementioned scenarious, blinding was not relevant for our study. Comparisons were performed automatically using statistical software, not influenced by the researcher.

Reporting for specific materials, systems and methods

We require information from authors about some types of materials, experimental systems and methods used in many studies. Here, indicate whether each material, system or method listed is relevant to your study. If you are not sure if a list item applies to your research, read the appropriate section before selecting a response.

Materials & experimental systems

n/a	Involved in the study	
☐	☒	Antibodies
☐	☒	Eukaryotic cell lines
☒	☐	Palaeontology and archaeology
☐	☒	Animals and other organisms
☒	☐	Clinical data
☒	☐	Dual use research of concern
☒	☐	Plants

Methods

n/a	Involved in the study	
☒	☐	ChIP-seq
☐	☒	Flow cytometry
☒	☐	MRI-based neuroimaging

Antibodies

Antibodies used

CD31-PECy7 BioLegend, Cat# 102523
CD45.2-FITC BioLegend, Cat# 104
CD11b-PerCP Biolegend, Cat# 101229
Ly6G/C-APC Thermo Fisher Scientific, Cat# 17-5931-82
NK1.1-APC BioLegend, Cat# 108710
CD3-FITC Thermo Fisher Scientific, Cat# 11-0032-82
CD140A-APC BioLegend, Cat# APA5
TCRb-APC780 Thermo Fisher Scientific, Cat# 47-5961-82
CD4-PerCP710 Thermo Fisher Scientific, Cat# 46-0042-82
CD4-FITC BioLegend, Cat# 100405
Foxp3 – APC Thermo Fisher Scientific, Cat# 17-5773-82
NK1.1-PE Thermo Fisher Scientific, Cat# 12-5941-82
TCRβ-PE Thermo Fisher Scientific, Cat# 12-5961-83
CD45-APC-Cy7 BD Biosciences, Cat# 560579
α-SMA-FITC Sigma-Aldrich, Cat# a2547

Rabbit anti-CD63 Santa Cruz Biotechnology, Cat# sc-15363
Mouse anti-CD63 Novus Biologicals®, Cat# NBP2-42225, H5C6
Goat anti-mCherry Biorbyt, Cat# orb116118
Chicken anti-mCherry abcam, Cat# ab205402
Chicken anti-GFP abcam, Cat# ab13970
Sheep anti-GFP BIO-RAD, Cat# 4745-1051
Rabbit anti-phiYFP Evrogen, Cat# AB603
Rabbit anti-mCherry and rat anti-mTFP antibodies were developed and kindly provided by Cai Laboratory, University of Michigan Medical School, Michigan, USA
Rabbit anti-CD31 abcam, Cat# ab28364
Rabbit anti-CD161 abcam, Cat# ab234107
Rabbit anti-CD4 abcam, Cat# ab183685
Rabbit anti-CD8 abcam, Cat# ab209775
Rabbit anti-Foxp3 abcam, Cat# ab215206
Mouse anti-CD68 abcam, Cat# ab31630
Mouse anti-Rab27a abcam, Cat# ab55667
Rabbit anti-EpCam abcam, Cat# ab71916
Rabbit anti-Syntenin abcam, Cat# ab19903
Mouse anti-Alix, Thermo Fisher Scientific Cat# MA1-83977
Goat anti-Apolipoprotein A1, Novusbio Cat# NB600-609
Rabbit anti-Cytochrome c, Santa Cruz Cat# sc-7159
Mouse anti-human CD63 BD Pharmingen Cat# 556019
Rat anti-mouse CD63 BD Pharmingen #564221
Rabbit anti-Megalin, abcam Cat# ab76969
Rabbit anti-Aquaporin-2, abcam Cat# ab199975
Rat anti-Podoplanin, abcam Cat# ab256559
Rabbit anti-Uteroglobin, abcam Cat# ab213203
Rabbit anti-TTF1, abcam Cat# ab76013

Anti-mouse Alexa-Fluor® 488 abcam, Cat# ab150105
Anti-mouse Alexa-Fluor® 594 abcam, Cat# ab150108
Anti-mouse Alexa-Fluor® 647 Thermo Fisher Scientific, Cat# A21235
Anti-chicken Alexa-Fluor® 633 Sigma, Cat# SAB4600127
Anti-sheep Alexa-Fluor® 488 Jackson ImmunoResearch, Cat# 713-545-003
Anti-rabbit Alexa-Fluor® 488 Jackson ImmunoResearch, Cat# 711-545-152
Anti-rabbit Alexa Fluor® 488 Thermo Fisher Scientific, Cat# A-11034
Anti-rabbit Alexa-Fluor® 546 Thermo Fisher Scientific, Cat# A10040
Anti-rat Alexa-Fluor® 488 Invitrogen, Cat# A21208
Anti-rat Alexa-Fluor® 594 Thermo Fisher Scientific, Cat# A21209
Anti-goat abcam, Cat# ab6741
Anti-chicken Sigma-Aldrich, Cat# A906
Anti-rabbit Cell Signalling, Cat# 7074
Anti-rat GenScript, Cat# A00167

The dilution used for each antibody is described in the manuscript methods section in an assay dependent fashion.

Validation

All antibodies have been validated in published studies (Cai et al., 2013, Nature Methods) and/or by the manufacturer when acquired from commercial vendors. We have included a validation in Supplementary Figure 4b.

Eukaryotic cell lines

Policy information about cell lines and Sex and Gender in Research

Cell line source(s)

Human pancreatic ductal adenocarcinoma cell lines: T3M4 (RCB Cat# RCB1021 kindly provided by Dr. Christoph Kahlert, Universitatsklinikum Carl Gustav Carusan der Technischen Universitat Dresden, Germany), PANC-1 (ATCC Cat# CRL-1469), BxPC-3 (ATCC Cat# CRL-1687), and MIA PaCa-2 (ATCC Cat# CRL1420). A mouse endothelial cell line: bEnd.3 (ATCC Cat# 2299).

Authentication

T3M4, PANC-1, BxPC-3 and MIAPaCa-2 were authenticated through STR profiling. bEnd.3 cell line was not authenticated.

Mycoplasma contamination

All cell lines tested negative for mycoplasma contamination.

Commonly misidentified lines
(See ICLAC register)

No commonly misidentified cell lines were used in this study.

Animals and other research organisms

Policy information about studies involving animals; ARRIVE guidelines recommended for reporting animal research, and Sex and Gender in Research

Laboratory animals

For the pancreas orthotopic surgery, 9-11 weeks old mice Rag2−/−Il2rg−/− (Cat# JAX:014593) were used.
Alleles from genetically engineered mouse models were acquired as follows:
Pdx1-Flp alleles were kindly provided by Dr. Dieter Saur, Technische Universität München, München, Germany and Dr. Michael Ostrowski, Department of Biochemistry and Molecular Biology, Medical University of South Carolina, Charleston, USA.
FSF-KRASG12D/+ and Trp53Frt/+ were kindly provided by Dr. Dieter Saur, Technische Universität München, München, Germany.
R26LSL-FLPoERT2/+ was kindly provided by Dr. David Goodrich, Roswell Park Cancer Institute, Buffalo, USA.
The PKT alleles (Ptf1a-Cre; LSL-KrasG12D/+; Tgfbr2loxP/loxP) were purchased from Jackson Laboratory: Ptf1atm1(cre)Hnak/RschJ (RRID:IMSR_JAX:023329); B6;129-Tgfbr2tm1Karl/J (RRID:IMSR_JAX:012603).
The KPC alleles (LSL-KrasG12D/+; LSL-Trp53R172H/; Pdx1-Cre) were purchased from Jackson Laboratory: B6.129S4-Krastm4Tyj/J (IMSR Cat# JAX:008179); 129S-Trp53tm2Tyj/J (IMSR Cat# JAX:008652) and B6.FVBTg(Pdx1-cre)6Tuv/J (IMSR Cat# JAX:014647).
Panc-CD63-mCherry and Panc-ExoBow mice were sacrificied between 8-11 weeks of age.
Pdx1-Flp Rab27aKO mice were sacrificied around 8 weeks of age.
KPC-ExoBow mice were sacrificied from 8-24 weeks of age (Pdx1Flp-KPC-ExoBow: around 17weeks, KPC-iExoBow: 17-24 weeks, Adeno-Flp-KPC-ExoBow: 8-12 weeks).
KPF CD63-mCherry mice were sacrificied between 15-21 weeks of age.
KPC mice analyzed had around 12 weeks of age.
PKTiRab27a cohort mice were sacrificied at humane endpoint between 6-13 weeks of age.
Details of animals and animal-derived materials were described in Methods. Mice were maintained under specificpathogen-free conditions in the animal facility of i3S Institute. The animal room has a controlled temperature (18-23°C), humidity (40-60%), and a 12 light/12 dark cycle.

Wild animals

This study did not involve wild animals.

Reporting on sex

For the pancreas orthotopic surgery, equal number of female and male Rag2−/−Il2rg−/− were used. For the genetically engineered mouse models, due to the high genetic complexity of the models developed, mice were used according to availability with no particular consideration for the sex of the animals.

Field-collected samples

This study did not involve field-collected samples.

Ethics oversight	All mice were housed under standard housing conditions at the i3S animal facility, and all animal procedures were reviewed and approved by the i3S Animal Welfare and Ethics Body and the animal protocol was approved by DGAV "Direção Geral de Alimentação e Veterinária".

Note that full information on the approval of the study protocol must also be provided in the manuscript.

Plants

Seed stocks	N/A
Novel plant genotypes	N/A
Authentication	N/A

Flow Cytometry

Plots

Confirm that:

☒ The axis labels state the marker and fluorochrome used (e.g. CD4-FITC).

☒ The axis scales are clearly visible. Include numbers along axes only for bottom left plot of group (a 'group' is an analysis of identical markers).

☒ All plots are contour plots with outliers or pseudocolor plots.

☒ A numerical value for number of cells or percentage (with statistics) is provided.

Methodology

Sample preparation	Pancreas and pancreatic tumors were minced with scalps and digested in digestion buffer (0,012% Dispase II Sigma-Aldrich D4693, 0,012% Collagenase Sigma-Aldrich C7657 in Hanks Balanced Salt Solution - HBSS 1X) for 10 min (pancreas) and 20 min (tumors) at 37ºC in agitation. The cell suspension was filtered using a 70-µm filter to obtain a single-cell suspension. Next, cells were incubated with Red Blood Cell lysis buffer for 5 min at room temperature (RT) and HBSS 1X was added in excess to stop the reaction. After centrifugation, a blocking step using blocking buffer was performed for 15min at RT. Finally, antibody staining was performed in FACS buffer (FBS 2% in HBSS 1X) for 30 min at 4ºC, followed by several washing steps. Prior to analysis samples were filtered through a 35µm cell strainer (Falcon).<br><br>Extracellular vesicles were mixed with 4 µm aldehyde/sulphate latex beads (Thermo Fisher Scientific ,A37304) in PBS1X for 45 min at RT with continuous rotation. Then, followed a quenching and blocking steps, primary antibody incubation and secondary antibody incubation, with the appropriate washing steps. For intracellular stainings, the extracellular vesicles were mixed with 4 µm aldehyde/sulphate latex beads (Thermo Fisher Scientific ,A37304) in PBS1X for 15 min at RT with continuous rotation, followed by onervight incubation at 4C. In the next day, followed a quenching and blocking, fixation and permeabilization steps, primary antibody incubation and secondary antibody incubation, with the appropriate washing steps.
Instrument	BD FACS Aria II Cell Sorter (BD Biosciences)<br>BD FACSCANTO II (BD Biosciences)<br>LSR Fortessa(BD Biosciences)<br><br>ImageStreamx MarkII Imaging Flow Cytometry (Luminex Amnis Image Stream Multispectral Imaging Flow Cytometer).
Software	FlowJo software (version 10, BD).
Cell population abundance	Sorting was not used in this study.
Gating strategy	FSC by SSC to remove debris; FSC-H by FSC-A to define single cells. Negative gating for viable cells. Positive populations were defined using not stained cells as reference. An example of the gating strategy is provided on the source data file. A detailed gating strategy is provided in the source data file.

☒ Tick this box to confirm that a figure exemplifying the gating strategy is provided in the Supplementary Information.
